# Effect of prostatic apex shape (Lee types) and urethral sphincter length in preoperative MRI on very early continence rates after radical prostatectomy

**DOI:** 10.1007/s11255-021-02809-7

**Published:** 2021-02-19

**Authors:** Mike Wenzel, Felix Preisser, Matthias Mueller, Lena H. Theissen, Maria N. Welte, Benedikt Hoeh, Clara Humke, Simon Bernatz, Boris Bodelle, Christoph Würnschimmel, Derya Tilki, Hartwig Huland, Markus Graefen, Frederik C. Roos, Andreas Becker, Pierre I. Karakiewicz, Felix K. H. Chun, Luis A. Kluth, Philipp Mandel

**Affiliations:** 1 Department of Urology, University Hospital Frankfurt, Goethe University, Frankfurt am Main, Germany; 2 Cancer Prognostics and Health Outcomes Unit, Division of Urology, University of Montreal Health Center, Montreal, QC Canada; 3 Department of Diagnostic and Interventional Radiology, University Hospital Frankfurt, Frankfurt am Main, Germany; 4 Martini-Klinik Prostate Cancer Center, University Hospital Hamburg-Eppendorf, Hamburg, Germany; 5 Department of Urology, University Hospital Hamburg-Eppendorf, Hamburg, Germany

**Keywords:** ORP, RARP, Lee type, functional outcome, PAD-test

## Abstract

**Purpose:**

To test the effect of anatomic variants of the prostatic apex overlapping the membranous urethra (Lee type classification), as well as median urethral sphincter length (USL) in preoperative multiparametric magnetic resonance imaging (mpMRI) on the very early continence in open (ORP) and robotic-assisted radical prostatectomy (RARP) patients.

**Methods:**

In 128 consecutive patients (01/2018–12/2019), USL and the prostatic apex classified according to Lee types A–D in mpMRI prior to ORP or RARP were retrospectively analyzed. Uni- and multivariable logistic regression models were used to identify anatomic characteristics for very early continence rates, defined as urine loss of ≤ 1 g in the PAD-test.

**Results:**

Of 128 patients with mpMRI prior to surgery, 76 (59.4%) underwent RARP vs. 52 (40.6%) ORP. In total, median USL was 15, 15 and 10 mm in the sagittal, coronal and axial dimensions. After stratification according to very early continence in the PAD-test (≤ 1 g vs. > 1 g), continent patients had significantly more frequently Lee type D (71.4 vs. 54.4%) and C (14.3 vs. 7.6%, *p* = 0.03). In multivariable logistic regression models, the sagittal median USL (odds ratio [OR] 1.03) and Lee type C (OR: 7.0) and D (OR: 4.9) were independent predictors for achieving very early continence in the PAD-test.

**Conclusion:**

Patients’ individual anatomical characteristics in mpMRI prior to radical prostatectomy can be used to predict very early continence. Lee type C and D suggest being the most favorable anatomical characteristics. Moreover, longer sagittal median USL in mpMRI seems to improve very early continence rates.

## Introduction

In the treatment of prostate cancer with radical prostatectomy for local tumor control, sufficient postoperative functional outcomes—mainly continence and potency—are of major importance to provide high quality of life in men [1–4]. In the recent years, several surgical techniques, such as nerve-sparing procedures or preservation of the “Full-Functional-Length-Urethra” (FFLU) have been investigated with the aim of improving early postoperative continence rates after open (ORP) and robotic-assisted radical prostatectomy (RARP) [5–9].

In shared decision making with prostate cancer patients regarding treatment choice of radical prostatectomy vs. radiation therapy, it is crucial to provide information and predict the postoperative probability of functional outcomes like early continence. Several risk factors for higher risk of postoperative incontinence as for example non-organ confined disease, age, non-nerve-sparing surgery, prostate volume or Gleason score have been previously described [10, 11]. Moreover, multiparametric magnetic resonance imaging (mpMRI) is nowadays frequently employed for prostate cancer diagnostics, staging and surgical planning purposes and also individual anatomical characteristics can be visualized and analyzed according to their influence on continence rates after radical prostatectomy [12]. For example, the urethral sphincter length measured in the preoperative mpMRI and also the variation of the prostatic apex shape, classified as four types as previously described by Lee et al. may yield the potential to predict continence rates [13–17]. Nonetheless, further validation of those predictors and interindividual anatomical characteristics is needed, since mpMRI quality improved in the recent years and RARP approach may allow better visibility of anatomical structures. Moreover, to the best of our knowledge the influence of these characteristics on very early continence, defined as urine loss in the validated PAD-test within 24 h after catheter removal, has never been investigated.

We addressed this void in analyzing our institutional radical prostatectomy database according to very early continence rates of the most recent patients who underwent mpMRI prior to ORP or RARP between 01/2018 and 12/2019. We hypothesized that very early postoperative continence rates differ according to the apex shape of the prostate, classified as Lee types A–D, as well as urethral sphincter length after ORP and RARP.

## Methods

### Study population

After approval of the local ethic committee, we identified all patients (*n* = 334) who underwent radical prostatectomy for biopsy confirmed prostate cancer at the University Hospital Frankfurt between 01/2018 and 12/2019 in the institutional radical prostatectomy database. Patients without preoperative mpMRI were excluded (*n* = 206). All surgeons in this patient cohort were experienced surgeons trained in high-volume prostate cancer centers. Radical prostatectomy was performed as ORP or RARP using FFLU-technique and frozen sections for nerve-sparing surgery, as previously described [6, 18].

### mpMRI prior to radical prostatectomy: Lee type definition and urethral sphincter length

mpMRI acquisition was performed as recommended (European Society of Urogenital Radiology ESUR guidelines) and previously described [19]. Moreover, according to the previous publication by Lee et al. and its value for continence prediction, prostatic apex shape in the mpMRI was classified as Lee type A–D in T2-weighted sequences [17]: Lee type A was defined as a prostatic apex overlapping the membranous urethra anteriorly and posteriorly. Lee type B and C were, respectively, defined as an overlap of the prostatic apex of the anterior or posterior membranous urethra. In addition, Lee type D was defined as no observed overlap of the prostatic apex over the membranous urethra in the mpMRI.

**Fig. 1:**
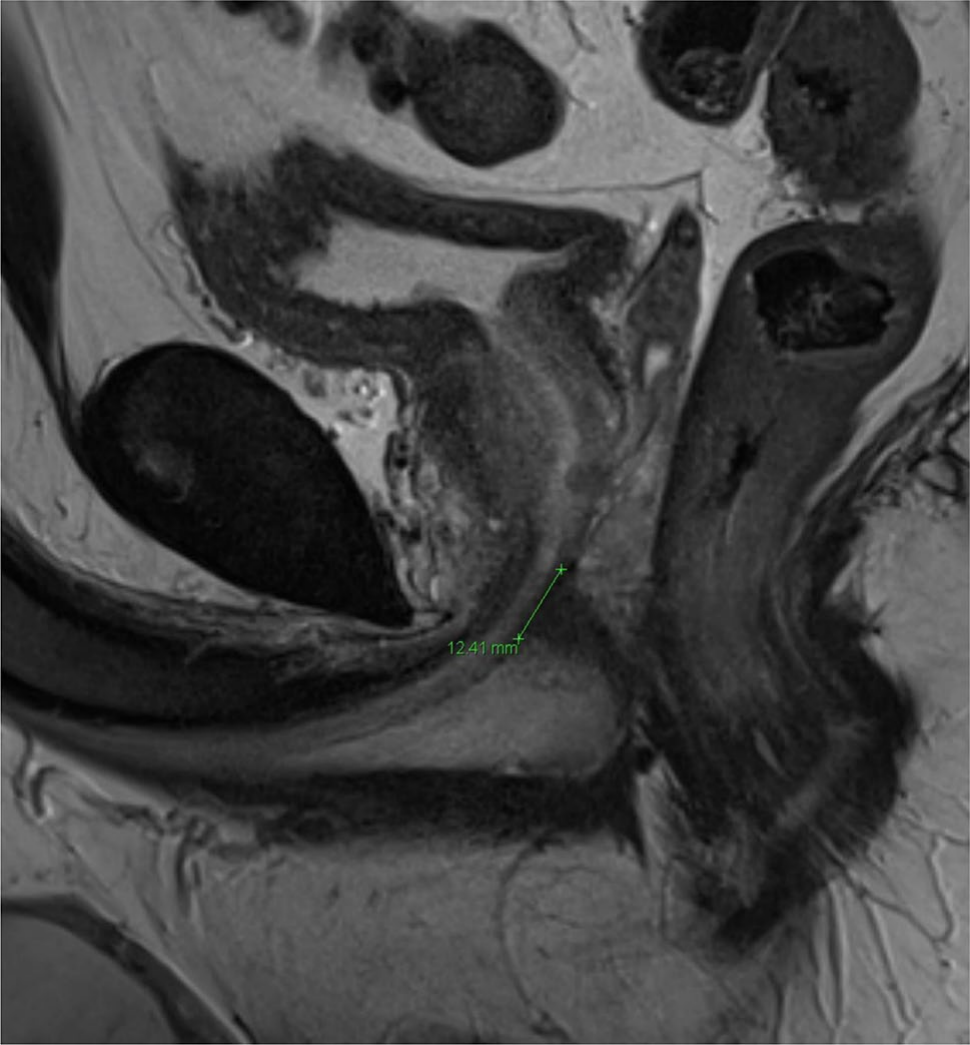
Urethral sphincter measurement in sagittal dimension in a multiparametric MRI prior to radical prostatectomy

Moreover, urethral sphincter length and diameter were measured (in millimeters [mm]) in sagittal, coronal and axial directions in preoperative mpMRI (Fig. 1). All mpMRI analyses were primarily performed by an experienced researcher with special training in uropathological imaging in a blinded fashion with regard to the study endpoint, supervised by a board-certificated radiologist.

### Continence rates after radical prostatectomy

Very early continence after ORP and RARP was defined as continence within 24 h after removal of the transurethral catheter. The catheter was removed after unsuspicious cystogram, routinely performed between the fifth to the seventh day after surgery. A standardized assessment of very early continence rates was performed in all patients with the PAD-test within 24 h after catheter removal, as previously described [10]. The PAD-test describes a comprehensible and validated test which measures the amount of involuntary urine loss while performing predefined physical activities in 1 h (e.g., coughing, walking, climbing stairs). Prior to the PAD-test, a pre-weighed collecting device was given to the patient and after physically activities, the collecting device was removed and reweighed. Very early continence was defined as a urine loss ≤ 1 g in the PAD-test [20].

### Statistical analyses

Descriptive statistics included frequencies and proportions for categorical variables. Means, medians and interquartile ranges (IQR) were reported for continuously coded variables. The Chi-square test was used for statistical significance in proportions’ differences. The *t* test and Kruskal–Wallis test examined the statistical significance of means’ and distributions’ differences.

Univariable and multivariable logistic regression models were fitted to test the relationship between Lee types A–D and urethral sphincter length in the preoperative mpMRI on the very early postoperative continence. Logistic regression models were set for the covariables age, prostate volume, pT-stage, nerve-sparing procedure and surgical approach (ORP vs. RARP). Statistically significant variables in univariable models were used for additional multivariable logistic regression models. All tests were two sided with a level of significance set at *p* < 0.05 and R software environment for statistical computing and graphics (version 3.4.3) was used for all analyses.

## Results

### Descriptive baseline characteristics

Between 01/2018 and 12/2019, 334 patients underwent radical prostatectomy for biopsy confirmed prostate cancer at the University Hospital Frankfurt. Of these, 128 patients had a preoperative mpMRI prior to radical prostatectomy and were included in the final analyses. In total, 76 (59.4%) patients underwent RARP vs. 52 (40.6%) ORP. Median age and median PSA were 66 (IQR: 60–70) and 8.0 ng/ml (IQR 6.0–11.5), respectively. In our cohort, 38 (29.7%), 16 (12.2%) and 29 (22.7%) patients inherited unfavorable characteristics such as high risk D’amico score, cT-stage ≥ 2c and Gleason Score 8–10 (ISUP 4–5), respectively. Neurovascular bundle preservation was performed on both sides in 92 (71.9%) and at one side in 24 (18.8%) patients. Overall, 49 (38.3%) patients had a very early continence with ≤ 1 g urine loss in the PAD-test. Further stratification was performed according to PAD-test ≤ 1 g vs. > 1 g (Table 1).

**Table 1:** Patient characteristics of 128 patients who underwent radical prostatectomy for biopsy confirmed prostate cancer between 01/2018 and 12/2019 at the University Hospital Frankfurt and received preoperative mpMRI, stratified by very early continence rates, classified by PAD-test (0–1 g vs. > 1 g)

Variable	Overall *n* = 128	PAD-test 0–1 g *n* = 49 (38.3%)	PAD-test > 1 g *n* = 79 (61.7%)	*p* value
Age, in years				
Median (IQR)	66 (60–70)	65 (60–68)	66 (60–72)	0.3
PSA, in ng/ml				
Median (IQR)	8.0 (6.0–11.5)	7.0 (6.0–10.0)	8.0 (6.0–14.0)	0.04
Length of urethral sphincter, sagittal, in mm				
Median (IQR)	15 (12–17)	15 (14–18)	15 (12–17)	0.03
Length of urethral sphincter, coronal, in mm				
Median (IQR)	15 (12–17)	15 (13–18)	15 (12–17)	0.1
Diameter of urethral sphincter, axial, in mm				
Median (IQR)	10 (9–11)	10 (9–11)	10 (9–11)	0.9
Diameter of urethral sphincter, coronal, in mm				
Median (IQR)	9 (8–10)	9 (8–10)	9 (8–10)	0.2
Prostate volume, in ml				
Median (IQR)	40 (30–50)	40 (30–45)	40 (29–50)	0.8
D’Amico score, *n* (%)				
Low	12 (9.4)	9 (18.4)	3 (3.8)	< 0.01
Intermediate	76 (59.4)	31 (63.3)	45 (57.0)	
High	38 (29.7)	8 (16.3)	30 (38.0)	
Missing information	2 (1.6)	1 (2.0)	1 (1.3)	
Lee type, *n* (%)				
A	21 (16.4)	3 (6.1)	18 (22.8)	0.03
B	16 (12.5)	4 (8.2)	12 (15.2)	
C	13 (10.2)	7 (14.3)	6 (7.6)	
D	78 (60.9)	35 (71.4)	43 (54.4)	
pT-stage, *n* (%)				
pT2	68 (53.1)	30 (61.2)	38 (48.1)	0.2
≥ pT3	60 (46.9)	19 (38.8)	41 (51.9)	
Surgical approach, *n* (%)				
ORP	52 (40.6)	13 (26.5)	39 (49.4)	0.02
RARP	76 (59.4)	36 (73.5)	40 (50.6)	
Nerve sparing, *n* (%)				
None	12 (9.4)	1 (2.0)	11 (13.9)	0.1
Unilateral	24 (18.8)	8 (16.3)	16 (20.3)	
Bilateral	92 (71.9)	40 (81.6)	52 (65.8)	
Surgical margin, *n* (%)				
Negative	88 (68.8)	37 (75.5)	51 (64.6)	0.3
Positive	40 (31.2)	12 (24.5)	28 (35.4)	

*IQR* interquartile range, *PSA* initial prostate-specific antigen

### mpMRI characteristics: urethral length and Lee types

Median length of the urethral sphincter was 15 mm (IQR 12–17), 15 mm (IQR 12–17) in the entire cohort in the sagittal and coronal dimension, respectively. Moreover, median axial and coronal diameter of the urethral sphincter were 9 mm (IQR 8–10) and 10 mm (IQR 9–11). Proportions of Lee type A, B, C and D were 21 (16.4%), 16 (12.5%), 13 (10.2%) and 78 (60.9%) in the entire cohort.

After stratification according to very early continence in the PAD-test (≤ 1 g vs. > 1 g), a significant longer median length of the urethral sphincter in sagittal dimension was observed in the group with a PAD-test ≤ 1 g vs. > 1 g (15 mm [IQR 14–18] vs. 15.0 [IQR 12–17, *p* = 0.03]. Conversely, no differences in the coronal and axial dimensions of the urethral length were observed between both groups (all *p* > 0.05). Furthermore, patients with a PAD-test ≤ 1 g after catheter removal had significantly more frequently Lee type D (71.4 vs. 54.4%) and C (14.3 vs. 7.6%, *p* = 0.03) than patients with a PAD-test > 1 g. Conversely, patients with a PAD-test > 1 g had significantly more frequently Lee type A (22.8 vs. 6.1%) and B (15.2. vs. 8.2%) in the mpMRI prior to radical prostatectomy than patients with a PAD-test ≤ 1 g (*p* = 0.03).

### PAD-test ≤ 1 g vs. PAD-test > 1 g

In univariable logistic regression analyses (Table 2), the length of the urethral sphincter in sagittal dimension (odds ratio [OR] 1.2), prostatic apex Lee type C (OR 7.0) and D (OR 4.9) and RARP (OR 2.7) were significant predictors for a very early continence in the PAD-test (all *p* < 0.05). In multivariable analyses, median length of the urethral sphincter in the sagittal dimension (OR 1.03), as well as Lee type C (OR 1.5) and D (OR 1.3) were independent predictors for very early continence, defined as PAD-test ≤ 1 g (all *p* < 0.05). Conversely, RARP or ORP had no influence on very early continence rates in multivariable logistic regression models (*p* > 0.05).

**Table 2:** Univariable and multivariable logistic regression models predicting very early continence after radical prostatectomy, defined as PAD-test 0–1 g

	Univariable analysis				Multivariable analysis			
	OR	CI 2.5%	CI 97.5%	*p* value	OR	2.5%	97.5%	*p* value
Length of urethral sphincter, sagittal, in mm	1.15	1.03	1.29	0.01	1.03	1.01	1.05	0.037
Length of urethral sphincter, coronal, in mm	1.12	1.00	1.27	0.06	–	–	–	–
Diameter of urethral sphincter, axial, in mm	1.02	0.81	1.28	0.9	–	–	–	–
Diameter of urethral sphincter, coronal, in mm	1.18	0.94	1.49	0.2	–	–	–	–
Lee type A	Ref (1.0)	–	–	–	–	–	–	–
B	2.00	0.38	11.74	0.4	1.15	0.85	1.55	0.4
C	7.00	1.47	41.67	0.02	1.53	1.10	2.11	0.01
D	4.88	1.50	22.06	0.02	1.27	1.01	1.59	0.04
Age	0.98	0.93	1.03	0.4	–	–	–	–
Prostate volume, in ml	0.99	0.96	1.01	0.3	–	–	–	–
Surgical approach ORP	Ref (1.0)	–	–	–	–	–	–	–
RARP	2.70	1.27	6.00	0.01	1.17	0.99	1.39	0.1
pT2 stage	Ref (1.0)	–	–	–	–	–	–	–
pT3–4	0.59	0.28	1.20	0.1	–	–	–	–
No nerve sparing	Ref (1.0)	–	–	–	–	–	–	–
Uni-/bilateral nerve sparing	7.76	1.44	144.3	0.1	–	–	–	–

*OR* Odds ratio, *CI* confidence interval, *PSA* prostate-specific antigen, *ORP* open radical prostatectomy, *RARP* robotic-assisted radical prostatectomy

## Discussion

We hypothesized that very early postoperative continence rates differ according to the apex shape of the prostate, classified as Lee types A–D, as well as the urethral sphincter length in mpMRI prior to surgery. We tested this hypothesis in our institutional radical prostatectomy database and made several noteworthy observations.

First, we made important observations according to the prostatic apex shape and the median urethral sphincter length. Specifically, the most common prostatic apex shape was Lee type D (61%), followed by A (16%), B (13%) and C (13%). Moreover, longest median urethral sphincter length was measured in sagittal dimension and was 15.1 mm (IQR 12.4–17.1). These observations are quite interesting, since geographical differences may exist regarding to the prostatic apex shape and the length of the median urethral sphincter. For example, in the first described classification of the prostatic apex shape overlapping the urethra by Lee et al., the majority of patients harbored Lee type A (38%) and the minority Lee type C (15%). Since Lee et al. demonstrated that the different anatomical shapes of the prostate apex influence urinary continence after radical prostatectomy, it is very important to investigate those geographical differences for comparisons and real-world application [17]. Moreover, also the median urethral length in sagittal dimension seems to differ across geographical areas. For example, in several studies and a meta-analysis by Mungovan et al., the median urethral length was mostly comparable to our findings in North American and European studies, while in Asian men, the median urethral sphincter length seemed to be shorter and ranged from 10.4 to 13.3 mm [14, 21, 22]. However, when determining Lee type and measuring urethral sphincter length, interobserver variability might be present and influence the results.

Second, we also made important observations after stratifying our cohort by PAD-test result ≤ 1 g vs. > 1 g. Specifically, patients with very early continence after catheter removal after radical prostatectomy harbored significantly more often none (Lee type D, 71 vs. 54%) or a posterior overlap (Lee type C, 14 vs. 8%) of the prostatic apex on the membranous urethra. Moreover, Lee type C and D were independent positive predictors for very early continence in multivariable logistic regression models. Comparing our findings with other studies, highest rates of urinary continence in the first publication by Lee et al. [17] were found in Lee type B or C 3 months after surgery. Since surgical techniques have impressively improved since the time of publication by Lee et al. and geographical differences in patients according to Lee types may exist as mentioned above, as well as the different analyzed timepoints for continence, the heterogeneity of both studies does not allow direct comparisons. It can be assumed that Lee type D (no prostatic apex overlap on the urethra) might be most favorable anatomical variant for surgeons to perform surgical techniques such as FFLU and this might reflect the high rates of very early postoperative continence [10, 22].

In addition, a significant longer median urethral length in the sagittal dimension in the mpMRI prior to radical prostatectomy was observed in patients with very early continence rates (PAD-test ≤ 1 g). Moreover, median sagittal urethral length was also an independent predictor for very early continence (OR 1.03) in multivariable logistic regression models after adjusting for the influence of surgical approach and techniques. These findings are very important since it can help clinicians to predict very early continence rates after catheter removal with the preoperative MRI and can provide a realistic preoperative continence prediction for the patients, despite the influence of for example, surgical approach, FFLU or nerve-sparing technique [5]. To the best of our knowledge, no previous study investigated the effect of Lee types and urethral sphincter length on very early continence outcomes, defined as continence measurement within 24 h after removal of the catheter after radical prostatectomy. In consequence, our data cannot be directly compared to other investigations. However, several studies reported a significant influence of the urethral length on continence rates, using different follow-up durations and continence definitions. For example, Kim et al. reported a significant influence of median urethral length on continence rates, defined as a PAD-test < 2 g, 3 months after surgery [21]. Moreover, Ko et al. reported a significant influence of a median urethral length > 14 mm on 30-day continence rates, defined as pad-free continence [23]. It is of note that since we used a stricter cutoff for the definition of continence (≤ 1 g in PAD-test) and the PAD-test was performed within 24 h after catheter removal, we believe that our findings even stronger emphasize and corroborate the effect of the urethral length on the urinary continence in an unbiased fashion.

Finally, we also made important observations between the comparison of ORP vs. RARP on continence rates. Patients with a PAD-test ≤ 1 g underwent more frequently RARP than ORP. However, after multivariable adjustment for differences in patient characteristics, the surgical approach did not influence very early continence rates, defined as PAD-test result of ≤ 1 g within 24 h after catheter removal. These findings are also important in the context of an often-stated argument of better visibility of anatomical structures with a RARP approach [24]. However, the same findings of no difference between RARP and ORP approach, after adjustment for age differences, were observed in a recently published comparison of a Martini-Klinik series with 10,790 radical prostatectomy patients regarding to continence rates 3 and 12 months after surgery [25].

Our study has several limitations and should first be interpreted in its retrospective design. Second, sample size limitations might impair statistical significance in some of the analyses. Third, even though all mpMRI were analyzed by an experienced specialist in urologic imaging who was supervised by a board-certificated radiologist interobserver variability cannot be ruled out. Moreover, only patients with an mpMRI and catheter removement during the in-hospital stay were included, which may have led to a selection bias. Fourth, both surgical approaches (ORP and RARP) represent methods with different learning curves and surgery was performed by different surgeons. Those factors may have influenced very early continence rates. Unfortunately, other endpoints with longer follow-up duration were not available. Prospective studies are needed to further validate or reject our findings.

Taken together, our findings demonstrate that the anatomical prostate apex shape of Lee type C and D in the mpMRI prior to surgery provided the best prediction for very early continence rates after catheter removal after radical prostatectomy. Moreover, our data also demonstrated that the median urethral length of the sphincter in sagittal dimension in mpMRI predicted very early continence rates. According to the comparison of ORP vs. RARP, no differences existed according to very early continence rates after consideration of Lee types and urethral length. These findings are important with regard to the increasing numbers of performed mpMRI prior to radical prostatectomy and should be considered by clinicians for patients’ information and surgical planning purposes.

## Data Availability

On request. Used software R statistics (version 3.6.1).
